# Morphofunctional Features of Glomeruli and Nephrons After Exposure to Electrons at Different Doses: Oxidative Stress, Inflammation, Apoptosis

**DOI:** 10.3390/cimb46110748

**Published:** 2024-11-06

**Authors:** Grigory Demyashkin, Sergey Koryakin, Mikhail Parshenkov, Polina Skovorodko, Matvey Vadyukhin, Zhanna Uruskhanova, Yulia Stepanova, Vladimir Shchekin, Artem Mirontsev, Vera Rostovskaya, Sergey Ivanov, Petr Shegay, Andrei Kaprin

**Affiliations:** 1 Department of Digital Oncomorphology, National Medical Research Centre of Radiology, 2nd Botkinsky Pass., 3, 125284 Moscow, Russia; 2 Laboratory of Histology and Immunohistochemistry, Institute of Translational Medicine and Biotechnology, I.M. Sechenov First Moscow State Medical University (Sechenov University), Trubetskaya St., 8/2, 119048 Moscow, Russia; misjakj@gmail.com (M.P.);; 3 Research and Educational Resource Center for Immunophenotyping, Digital Spatial Profiling and Ultrastructural Analysis Innovative Technologies, Peoples’ Friendship University of Russia (RUDN University), Miklukho-Maklaya St., 6, 117198 Moscow, Russia; 4 Department of Urology and Operative Nephrology, Peoples’ Friendship University of Russia (RUDN University), Miklukho-Maklaya St., 6, 117198 Moscow, Russia

**Keywords:** electron irradiation, kidney, radiation nephropathy, tubulointerstitial fibrosis

## Abstract

Kidney disease has emerged as a significant global health issue, projected to become the fifth-leading cause of years of life lost by 2040. The kidneys, being highly radiosensitive, are vulnerable to damage from various forms of radiation, including gamma (γ) and X-rays. However, the effects of electron radiation on renal tissues remain poorly understood. Given the localized energy deposition of electron beams, this study seeks to investigate the dose-dependent morphological and molecular changes in the kidneys following electron irradiation, aiming to address the gap in knowledge regarding its impact on renal structures. The primary aim of this study is to conduct a detailed morphological and molecular analysis of the kidneys following localized electron irradiation at different doses, to better understand the dose-dependent effects on renal tissue structure and function in an experimental model. Male Wistar rats (*n* = 75) were divided into five groups, including a control group and four experimental groups receiving 2, 4, 6, or 8 Gray (Gy) of localized electron irradiation to the kidneys. Biochemical markers of inflammation (interleukin-1 beta [IL-1β], interleukin-6 [IL-6], interleukin-10 [IL-10], tumor necrosis factor-alpha [TNF-α]) and oxidative stress (malondialdehyde [MDA], superoxide dismutase [SOD], glutathione [GSH]) were measured, and morphological changes were assessed using histological and immunohistochemical techniques (TUNEL assay, caspase-3). The study revealed a significant dose-dependent increase in oxidative stress, inflammation, and renal tissue damage. Higher doses of irradiation resulted in increased apoptosis, early stages of fibrosis (at high doses), and morphological changes in renal tissue. This study highlights the dose-dependent effects of electrons on renal structures, emphasizing the need for careful consideration of the dosage in clinical use to minimize adverse effects on renal function.

## 1. Introduction

Chronic kidney disease (CKD) has become a significant global health issue, now recognized as the seventh-leading cause of death worldwide, with its impact continuing to grow over the past two decades [1,2]. By 2040, CKD is projected to rise to the fifth-leading cause of years of life lost (YLL) globally [3]. In addition to its high morbidity and mortality, CKD often develops as a long-term consequence of therapeutic interventions, particularly radiation therapy (RT). The kidneys, being highly radiosensitive, are especially susceptible to radiation-induced damage, which can result in both acute and chronic renal dysfunction [4]. Notably, approximately 23% of patients receiving abdominal or pelvic radiotherapy experience radiation nephropathy (RN), which can progress to CKD in a significant number of cases [5,6]. Given the high incidence of CKD following radiotherapy, understanding the specific effects of electron radiation on renal tissues becomes essential, as it could offer a safer therapeutic alternative that minimizes radiation-induced kidney damage, ultimately improving the quality of life for cancer survivors.

While the effects of gamma and X-ray radiation on kidney structures have been extensively studied, with well-documented early morphofunctional changes in the glomerular apparatus and nephrons, as well as late-stage fibrosis [4,5,6], the impact of electron radiation on kidney tissues remains largely unexplored. Most existing studies focus on the effects of gamma and X-ray radiation, leaving a significant gap in our understanding of how electron radiation impacts renal structures. This gap is especially notable given the potential differences in biological effects between these forms of radiation. Unlike gamma rays, which penetrate deeply and interact with tissues over a broader area [7], electrons have a more localized energy deposition, potentially offering a more targeted and less damaging alternative for medical applications [8]. This limited penetration depth and focused energy deposition of electron beams provide a unique opportunity to study the dose-dependent effects on renal morphology and function, with the potential to reduce the collateral damage often associated with other forms of radiation therapy.

Radiation nephropathy (RN) is a serious and potentially debilitating condition resulting from exposure to ionizing radiation, particularly in the context of radiation therapy (RT) for malignant tumors in the abdominal and pelvic regions, where incidental renal exposure is often unavoidable [9,10,11]. Despite significant advancements in RT techniques, including stereotactic radiotherapy (SRT) and intensity-modulated radiotherapy (IMRT), the challenge of precisely targeting tumors while minimizing collateral damage to renal tissues remains substantial [12,13,14]. Existing data on the effects of radiation therapy on renal function indicate that irradiation can cause cellular damage in all components of the kidney, including glomeruli, blood vessels, tubular epithelium of the nephron, and interstitial tissue [15,16,17]. Among these, the most pronounced morphological changes have been observed in the endothelial cells of the glomeruli, which often detach from the basement membranes [10].

At the molecular level, RN is primarily driven by the induction of DNA double-strand breaks (DSBs) by ionizing radiation, which activates the DNA damage response (DDR) pathways [18]. While these pathways aim to repair the damage, their capacity can be overwhelmed, leading to genomic instability, cellular senescence, and chronic inflammation—key processes in the development of progressive renal fibrosis and CKD. Oxidative stress plays a critical role in this process, with an imbalance between reactive oxygen species (ROS) and antioxidant defenses exacerbating cellular damage and sustaining a pro-fibrotic environment within renal tissues [19]. This chronic oxidative stress contributes to the replacement of functional renal tissue with fibrotic scar tissue, which is a hallmark of chronic radiation nephropathy [20].

Simultaneously, the release of free radicals into the systemic circulation amplifies oxidative stress in other organs, prompting the body to upregulate the expression of natural antioxidants such as superoxide dismutase, glutathione, catalase, and others [21]. However, the increase in ROS levels, coupled with the diminished efficacy of these protective mechanisms, exacerbates cellular damage, leading to apoptosis or necrosis [22,23]. As a result, there is an elevated production of pro-inflammatory cytokines like IL-1, IL-6, and TNF-α in both blood and tissues, which further increases endothelial permeability and enhances the production of cyclooxygenase-2 (COX-2), triggering apoptotic signaling pathways [24]. This inflammatory response not only expands the affected area but also indirectly amplifies radiation-induced cellular damage. The extent of stromal vascular response modulation is closely linked to the production of IL-1, IL-6, and TNF-α, which play a critical role in mitigating the destruction of cellular structures.

Given the limited data on electron irradiation and its potential as a safer alternative to conventional radiation therapy, this study aims to fill this gap by elucidating the molecular and cellular changes induced by different doses of electron irradiation in renal tissues. The aim is to further our understanding of the underlying mechanisms of radiation nephropathy and to identify potential therapeutic targets to mitigate renal damage. The results of this study may draw the attention of the scientific community to this topic and provide valuable insights that may lead to the wider adoption of electron-based therapies in clinical practice, especially when minimizing damage to surrounding healthy tissues is of paramount importance and also when operating on the kidney itself.

## 2. Research Objective

### Aim of the Study

To conduct a comprehensive morphological and molecular assessment of the kidneys following localized electron irradiation at varying doses, with the objective of elucidating the dose-dependent effects on renal tissue structure and function in an experimental model (experimental study).

## 3. Material and Methods of Research

### 3.1. Experimental Animals

Male Wistar rats (220 ± 20 g; 9–10 weeks; *n* = 75) were housed in a controlled vivarium environment, maintained at a stable temperature of 22–23 °C with a 12 h light/dark cycle (12L:12D). The humidity was regulated between 40 and 60%, and the rats had free access to standard laboratory chow and water ad libitum. The animals were housed in pairs within plastic cages, lined with absorbent material (rice husk) to provide suitable nesting material and to reduce the stress associated with solitary confinement, which could influence their behavior and physiological responses.

Regarding the age of the rats (we used 9–10-week-old male Wistar rats), as they represent a stage of early adulthood in rodents. This choice allowed us to assess radiation-induced effects during a phase of high metabolic activity without the potential variability introduced by aging-related processes. As for the use of only male rats, this decision was made to avoid the hormonal fluctuations associated with the estrous cycle in females, which could have introduced additional variability in the study outcomes.

### 3.2. Experimental Design

**Figure 1:**
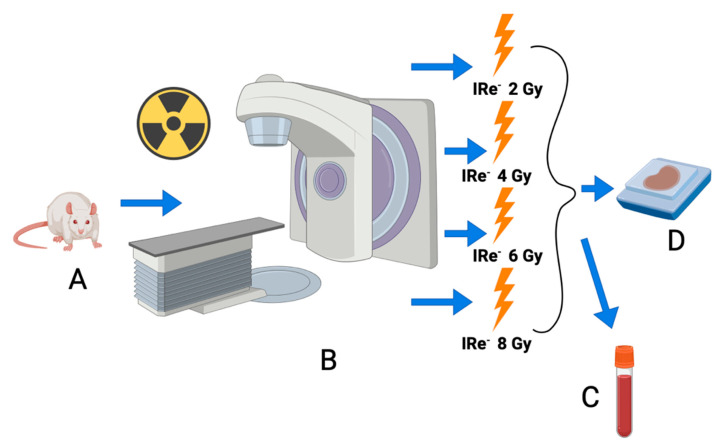
Design of the experiment. Special notations: (**A**)—Male Wistar rats (9–10 weeks old) were randomly assigned to five groups after a 7-day quarantine period. These groups included one control group (intact) and four experimental groups, each receiving a different dose of electron irradiation (2 Gy, 4 Gy, 6 Gy, and 8 Gy) targeted at the abdomino-pelvic region; (**B**)—irradiation was performed using a NOVAC-11 pulsed electron accelerator. Specific doses were administered with careful shielding to protect surrounding tissues; (**C**)—following irradiation, blood samples were collected from the animals for biochemical analysis (7 days post-irradiation). The evaluation of blood biochemical parameters was conducted according to the established research methodology; (**D**)—morphological examinations and organ homogenate studies were performed post-irradiation, following the procedures detailed in the research methodology (7 days post-irradiation).

The rats were divided into groups according to the design of the experiment (Figure 1): control group/intact (I; *n* = 15) and four experimental groups (II–V; *n* = 15 per group) based on the dose of single local electron irradiation of the abdominal–pelvic region, specifically targeting the kidney area: Group II received 2 Gy, Group III received 4 Gy, Group IV received 6 Gy, and Group V received 8 Gy.

Animals of all groups (I–VI) were removed from the experiment by administration of high doses of anesthetic (ketamine at a dose of 50 mg/kg intramuscularly and xylazine at a dose of 5 mg/kg intraperitoneally) on the 7th day.

### 3.3. Kidney Irradiation Model

Irradiation of the animals was performed using a NOVAC-11 pulsed electron accelerator (S.I.T. Sordina IORT Technologies S.P.A., Vicenza, Italy) at the Department of Radiation Biophysics of the A.F. Tsyb Medical Radiological Research Center. This device generates an electron beam with adjustable energy and collimation. For this experiment, the following parameters were selected: 10 MeV energy, 9 Hz frequency, and a collimation of Ø 40 mm. The dose rate was 1.5 Gy/min, and the irradiation time was approximately 1 min and 20 s for the 2 Gy dose, 2 min and 40 s for the 4 Gy dose, 4 min for the 6 Gy dose, and 5 min and 20 s for the 8 Gy dose. These settings allowed for precise and safe irradiation of the targeted kidney area in the rats, ensuring minimal collateral damage to surrounding tissues.

**Figure 2:**
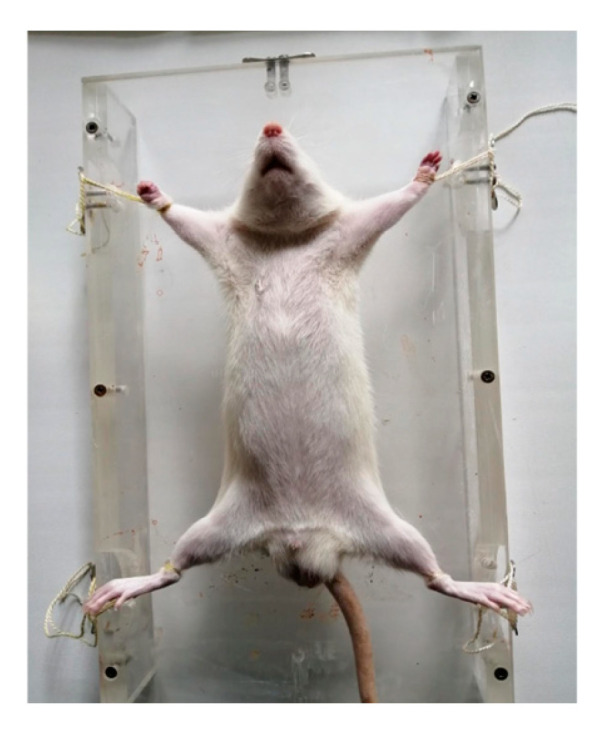
Specialized patented restraint devices (sleds), developed by the Laboratory of Radiation Pathomorphology of the A.F. Tsyb Medical Radiological Research Center.

Prior to irradiation, the rats in the experimental groups were sedated with a single intramuscular injection of ketamine (Alfasan International B.V., Woerden, The Netherlands; 50 mg/kg) and xylazine (Alfasan International B.V., The Netherlands; 5 mg/kg). The anesthetized animals were positioned individually on an examination table in a prone position with their limbs spread outwards, ensuring optimal access to the area under investigation (the kidneys). To maintain the animals’ immobility during the procedure, specialized patented restraint devices (sleds) were used (Figure 2).

To protect other parts of the body from radiation exposure, particularly the heart and lungs, shielding was applied to the areas outside the irradiation zone. For maximum precision, the tube was directed at the target area so that its end was no more than two millimeters from the skin, positioned strictly perpendicular to the surface.

The shielding material used was a 2 mm thick lead plate. Lead was chosen due to its high atomic number and density, which makes it one of the most effective materials for blocking ionizing radiation, particularly in electron and photon therapies. The lead shielding was cut to match the size and shape of the animals, ensuring it covered regions such as the thoracic cavity (to protect the heart and lungs) and lower abdomen while leaving the kidney area fully exposed. To prevent movement of the shielding during the irradiation process, the lead sheets were securely fastened to the experimental table using an adjustable clamp system. Additionally, the animals were positioned using a restraint device that minimized their movement during the procedure, ensuring that both the animal and the shielding remained in place. This method allowed us to maintain high precision in directing the electron beam to the intended kidney region while preventing unnecessary radiation exposure to other organs, which could have compromised the health of the animals or skewed the study results.

The appearance and weight of the animal (absolute—in grams and relative—in relation to body weight, in %), and the size and condition of the kidney parenchyma on section were assessed.

Animal welfare was a primary concern throughout the experiment. Every effort was made to minimize discomfort and stress, ensuring that the procedure was conducted in a humane and ethical manner.

### 3.4. Euthanasia and Organ Collection

Rats were humanely euthanized 7 days after irradiation by administering a high dose of ketamine (50 mg/kg intramuscularly) and xylazine (5 mg/kg intraperitoneally) to induce deep anesthesia, followed by exsanguination. This timepoint was selected to assess both acute and progressive changes in renal tissue. Following euthanasia, the kidneys were promptly excised and prepared for further analysis.

### 3.5. Assessment of Inflammatory Markers

The levels of cytokines IL-1β, IL-6 (Bender MedSystems, Vienna, Austria), IL-10 (Abcam, Eugene, OR, USA), and TNF-α (Assaypro, MO, USA, St. Charles, MO, USA) were measured in the serum of the animals. These measurements were conducted using commercial ELISA kits, following the manufacturer’s instructions.

Blood samples were collected 24 h before (basal point) and 7 days after irradiation from the retro-orbital sinus. The amount of blood collected was approximately 1.0–1.5 mL per animal, ensuring sufficient volume for biochemical and cytokine analysis while minimizing distress to the animals. After collection, the blood samples were allowed to clot at room temperature and were then centrifuged at 3000 rpm for 12 min to separate the serum, which was stored at −80 °C until analysis. All procedures were performed in strict accordance with institutional ethical guidelines for animal care.

### 3.6. Assessment of Oxidative Stress Markers

Kidney homogenate was prepared by homogenizing 1 g of tissue in 4.5 mL of cold potassium buffer (pH 7.4). The mixture was then centrifuged at 13,000 rpm for 10 min at 4 °C. The resulting supernatant was stored at −80 °C for further analysis. Levels of malondialdehyde (MDA), a biomarker of lipid peroxidation, as well as superoxide dismutase (SOD) and glutathione (GSH) in the kidney homogenate, were evaluated using ELISA kits (Lifespan Biosciences, Lynnwood, WA, USA).

Kidney samples were collected immediately after euthanasia, which occurred 7 days post-irradiation.

### 3.7. Morphological Block

After extraction, the appearance of the kidneys and the condition of the parenchyma on the cut (blood filling, inflammatory changes, atrophy, etc.) were assessed, weighed (weight in grams), and measured. Then, they were cut parallel to the sagittal plane every 2 mm, fixed in a solution of neutral formalin; after insertion (apparatus for histological tissue guiding, Leica Biosystems, Nussloch, Germany), they were embedded in paraffin blocks, from which serial sections (3 μm thick) were prepared, dewaxed, dehydrated, and stained with Mayer’s hematoxylin and eosin.

Quantification of interstitial fibrosis was performed using computer morphometry on kidney preparations stained with Masson’s trichrome.

Morphological analysis was carried out in 10 randomly selected fields of view of the microscope at a magnification of ×100 and ×400 in 4 random sections from each sample, moving the slides at equal intervals along the *X* and *Y* axes.

Computer morphometry was performed using the ImageJ 1.51 open-source computerized image analysis system to evaluate the results of the immunohistochemical reaction and to determine the area of positively stained objects in the field of view. For this, micrographs of 10 randomly selected fields of view were carried out at a magnification of ×200 on micro preparations with an IHC reaction performed with the corresponding primary antibodies. Then, the resulting image was automatically normalized and transferred from the 24-bit color picture (RGB) mode to the 256 grayscale mode, and the objects were binarized with a given sensitivity level. When processing micrographs by the image analysis system, binary objects with a brightness level > 150 were cut off and the total area of positively colored objects was determined from the total area of micrographs.

The assessment of the degree of fibrosis was carried out in points, taking into account the area and optical density (staining of fibers according to Masson) in relative units: “0”—absent; “1”—weak (0–0.3; <25%); “2”—moderate (0.3–0.6; 25–50%); “3”—strong (0.6–0.9; 50–75%); “4”—pronounced (>0.9; >75%).

The assessment of the degree of renal glomerular hypertrophy was carried out in points, considering their diameter in relative units: “0”—the norm (≤120 µm); “1”—increased (≥120 microns).

Evaluation of focal necrosis of the vascular glomerulus and necrosis of the epithelium of the tubules of the nephron was made in points from 0 to 3: 0—no changes; 1—1/3 of the affected area; 2—2/3 of the affected area; 3—>2/3 of the affected area. All criteria for assessing renal histopathological changes are summarized in Table 1.

### 3.8. TUNEL Analysis

The right kidney from each rat was processed for TUNEL analysis on 3 µm paraffin sections. Antigen retrieval was carried out by boiling the sections in 10 mM citrate buffer (pH 6.0) for 12 min to unmask the epitopes. Following antigen retrieval, the sections were fixed in 4% paraformaldehyde (pH 7.4) at −20 °C for 3 min to preserve tissue integrity. After fixation, the sections were thoroughly washed four times with phosphate-buffered saline (PBS) to remove any residual fixative. The tissue sections were then permeabilized with 0.1% Triton X-100 to ensure optimal penetration of the TUNEL reagent. The TUNEL assay was performed by incubating the permeabilized sections with a reagent containing terminal deoxynucleotidyl transferase (TdT) and fluorescein isothiocyanate (FITC)-labeled dUTP, which labels fragmented DNA indicative of apoptosis. After completing the TUNEL assay, the sections were stained with 4′,6-diamidino-2-phenylindole (DAPI) to label the nuclei, facilitating the visualization of all cellular nuclei within the tissue sections. DAPI staining was carried out by incubating the sections in a DAPI solution following the TUNEL assay and subsequent washes in PBS. Finally, the kidney sections were mounted in PBS and analyzed under a fluorescence microscope equipped with UV light to visualize both FITC-labeled apoptotic DNA fragments and DAPI-stained nuclei. All morphometric assessments were conducted in a blinded manner by at least five independent observers to ensure unbiased evaluation.

### 3.9. Immunohistochemistry (IHC) Staining

For immunohistochemical (IHC) analysis of caspase-3 expression in renal tissue, 3 µm thick paraffin-embedded kidney sections were prepared. Following deparaffinization, the sections were treated with a 0.3% hydrogen peroxide solution in methanol for 30 min to block endogenous peroxidase activity. Heat-induced antigen retrieval was performed in a citrate buffer (pH 6.0) using an autoclave for 20 min. The sections were then incubated with primary monoclonal antibodies against caspase-3 (clone 74T2; Thermo Fisher Scientific, Waltham, MA, USA) for 12 h at 4 °C. Secondary antibody detection was carried out using the HiDef Detection™ HRP Polymer system (Cell Marque, Waltham, MA, USA), which employs Anti-Rabbit/Mouse IgG conjugated with horseradish peroxidase (HRP). The immunoreaction was visualized using a DAB (3,3′-diaminobenzidine) substrate kit, resulting in a brown color indicative of caspase-3 positive staining. Cell nuclei were counterstained with Mayer’s hematoxylin solution. The number of caspase-3 immunopositive cells (brown-stained cytoplasm and/or nuclei) was quantified in 10 randomly selected fields of view at ×40 magnification. The percentage of caspase-3 positive cells was calculated for each sample.

### 3.10. Statistical Analysis

The data obtained from the calculations were processed using SPSS 12 for Windows (IBM Analytics, Austin, TX, USA). Results are expressed as mean ± SD (standard deviation). The Shapiro–Wilk test was used to assess the normality of the data distribution. For comparisons between study groups with non-normal distributions, the Kruskal–Wallis test followed by Dunn’s post-hoc test was applied. Multiple comparisons were performed using the Mann–Whitney U test. A *p*-value ≤ 0.05 was considered statistically significant.

## 4. Results

**Figure 3:**
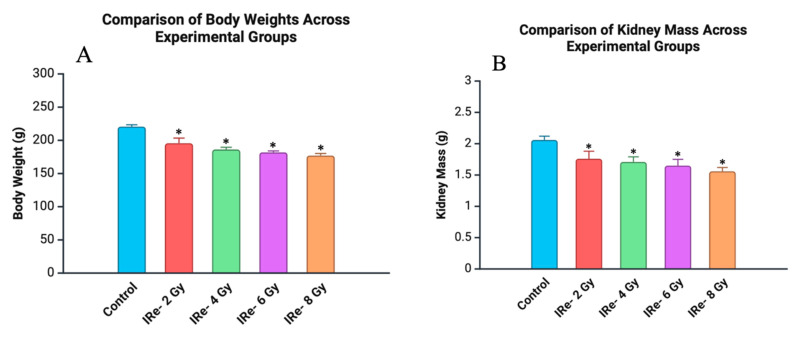
Comparison of body weight and kidney mass across experimental groups measured at 7 days post-irradiation. All data are presented as mean ± SD. Statistically significant differences are indicated by symbols: *—comparison with control group (*p* < 0.05). (**A**) Body weight of animals in experimental groups: the body weight of animals decreased progressively with increasing doses of electron irradiation, with the most significant reduction observed at 8 Gy. (**B**) Kidney mass in experimental groups: kidney mass showed a dose-dependent decrease, with the highest reduction occurring at 8 Gy compared to the control group.

The body weight of animals in all experimental groups decreased significantly compared to the control group, with the degree of reduction correlating with the dose of electron irradiation (Figure 3A). In the group exposed to 2 Gy of irradiation, body weight decreased by 11.0% from the control group (*p* < 0.05). As the irradiation dose increased to 4 Gy, body weight showed a 14.6% reduction compared to the control (*p* < 0.05). The 6 Gy group exhibited a 16.6% decrease in body weight from the control group (*p* < 0.05). The most significant reduction in body weight was observed in the 8 Gy group, with a 17.9% decrease relative to the control group (*p* < 0.05).

The kidney mass of animals in all experimental groups also decreased significantly compared to the control group, with the reduction correlating with the dose of electron irradiation (*p* < 0.05) (Figure 3B). In the group exposed to 2 Gy of irradiation, kidney mass decreased by 14.6% from the control group (*p* < 0.05). As the irradiation dose increased to 4 Gy, kidney mass showed a 17.0% reduction compared to the control (*p* < 0.05). The 6 Gy group exhibited a 19.9% decrease in kidney mass from the control group (*p* < 0.05). The most significant reduction in kidney mass was observed in the 8 Gy group, with a 21.4% decrease relative to the control group (*p* < 0.05).

### 4.1. Assessment of Inflammatory Markers

The assessment of inflammatory markers revealed a significant increase in cytokine levels across all irradiated groups compared to the control, with the magnitude of increase correlating with the dose of electron irradiation.

**Figure 4:**
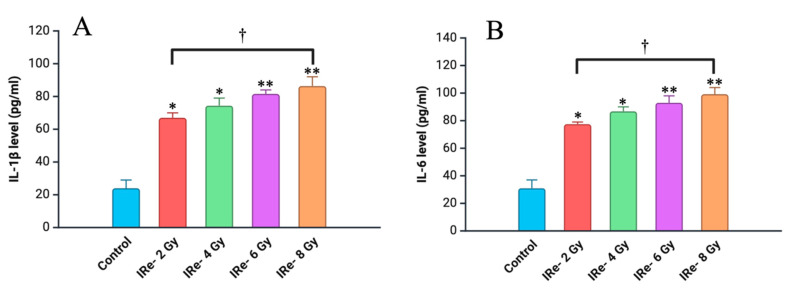

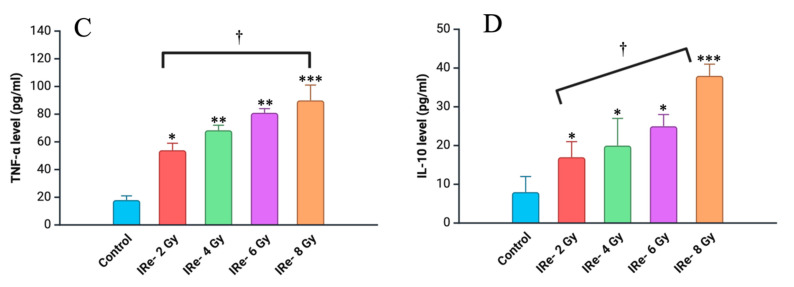
Levels of different cytokines in blood of experimental groups measured at 7 days post-irradiation: (**A**)—data for IL-1β; (**B**)—data for IL-6; (**C**)—data for TNF-α; (**D**)—data for IL-10. Data are presented as mean ± SD. Experimental groups are numbered according to the study design. Statistically significant differences are indicated by symbols: *—comparison with control group (*p* < 0.05); **—comparison with control group (*p* < 0.01); ***—comparison with control group (*p* < 0.001); †—comparison between Group II (2 Gy) and Group IV (8 Gy) (*p* < 0.01).

In the group exposed to 2 Gy, the level of IL-1β increased by approximately 2.8 times compared to the control group (*p* < 0.05). As the irradiation dose increased, IL-1β levels showed a further elevation. The group exposed to 4 Gy exhibited a 3.1-fold increase (*p* < 0.05), while the 6 Gy group demonstrated a 3.4-fold increase (*p* < 0.01). The most significant rise was observed in the group exposed to 8 Gy, where IL-1β levels increased by 3.6 times relative to the control (*p* < 0.01). These findings indicate a clear dose-dependent response, with higher doses of irradiation resulting in more pronounced inflammatory responses (Figure 4A).

Similarly, IL-6 levels in the blood showed a dose-dependent increase following irradiation. The 2 Gy group exhibited a 2.5-fold increase compared to the control group (*p* < 0.05). The group receiving 4 Gy showed a 2.7-fold increase (*p* < 0.05), and in the 6 Gy group, IL-6 levels were elevated by 2.9 times (*p* < 0.01). The 8 Gy group experienced the highest increase in IL-6 levels, which were 3.3 times higher than those in the control group (*p* < 0.01) (Figure 4B).

TNF-α levels also rose significantly across the irradiated groups. In the 2 Gy group, TNF-α was elevated by 3.0 times relative to the control (*p* < 0.05). The 4 Gy group showed a 3.8-fold increase (*p* < 0.01), while the 6 Gy group exhibited a 4.5-fold rise (*p* < 0.01). The highest levels of TNF-α were recorded in the 8 Gy group, with a 5.0-fold increase compared to the control (*p* < 0.001). This steep rise in TNF-α with increasing doses highlights the severe inflammatory response triggered by higher levels of electron irradiation (Figure 4C).

IL-10, an anti-inflammatory cytokine, showed an increase across all experimental groups in response to electron irradiation (Figure 4D). In the group exposed to 2 Gy, IL-10 levels increased by 2.25 times compared to the control. As the dose of irradiation increased to 4 Gy, IL-10 levels rose by 2.6 times. The 6 Gy group exhibited an increase of 2.9 times, while the most significant change was observed in the 8 Gy group, where IL-10 levels increased by 3.8 times.

### 4.2. Assessment of Oxidative Stress Markers

**Figure 5:**
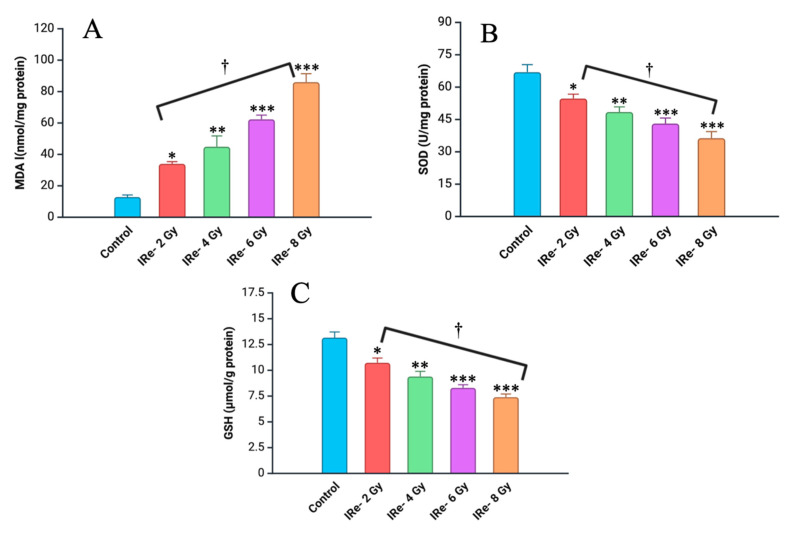
Levels of different markers of oxidative stress in kidney homogenate of experimental groups (7 days post-irradiation): (**A**)—data for MDA; (**B**)—data for SOD; (**C**)—data for GSH. Data are presented as mean ± SD. Experimental groups are numbered according to the study design. Statistically significant differences are indicated by symbols: *—comparison with control group (*p* < 0.05); **—comparison with control group (*p* < 0.01); ***—comparison with control group (*p* < 0.001); †—comparison between group II (2 Gy) and group IV (8 Gy) (*p* < 0.01).

Assessment of oxidative stress markers in kidney tissue homogenates after single-dose electron irradiation revealed significant changes in all experimental groups compared to the control. The level of malondialdehyde (MDA), a marker of lipid peroxidation, increased markedly in the irradiated groups, while the activity of superoxide dismutase (SOD) and glutathione (GSH) decreased significantly as the irradiation dose increased (Figure 5).

In the group exposed to 2 Gy of irradiation, MDA levels exhibited a significant increase, rising 2.6-fold compared to the control group (*p* < 0.05). This increase was more pronounced in the 4 Gy group, where MDA levels rose by 3.7-fold relative to the control (*p* < 0.01). The most substantial elevations in MDA were observed in the higher-dose groups, with levels increasing by 4.8-fold in the 6 Gy group (*p* < 0.001) and 6.7-fold in the 8 Gy group (*p* < 0.001), indicating a clear dose–response relationship.

Conversely, superoxide dismutase (SOD) activity demonstrated a significant dose-dependent reduction across the irradiated groups compared to the controls. In the 2 Gy group, SOD activity decreased by 19% (*p* < 0.05). This reduction was further exacerbated in the 4 Gy group, with a 29% decrease in SOD activity (*p* < 0.01). The 6 Gy and 8 Gy groups exhibited the most pronounced reductions, with SOD activity declining by 27.6% (*p* < 0.001) and 42.4% (*p* < 0.001), respectively.

Similarly, glutathione (GSH) levels were significantly diminished in a dose-dependent manner across all irradiation groups compared to the controls. A 19% reduction in GSH levels was observed in the 2 Gy group (*p* < 0.05), while a more substantial decrease of 29% was noted in the 4 Gy group (*p* < 0.01). The most significant reductions were seen in the 6 Gy and 8 Gy groups, where GSH levels decreased by 37.1% (*p* < 0.001) and 44.7% (*p* < 0.001), respectively.

These findings underscore the significant dose-dependent oxidative damage induced by electron irradiation, characterized by increased lipid peroxidation and a concurrent depletion of key antioxidant defenses in renal tissues.

### 4.3. Morphological Block

**Figure 6:**
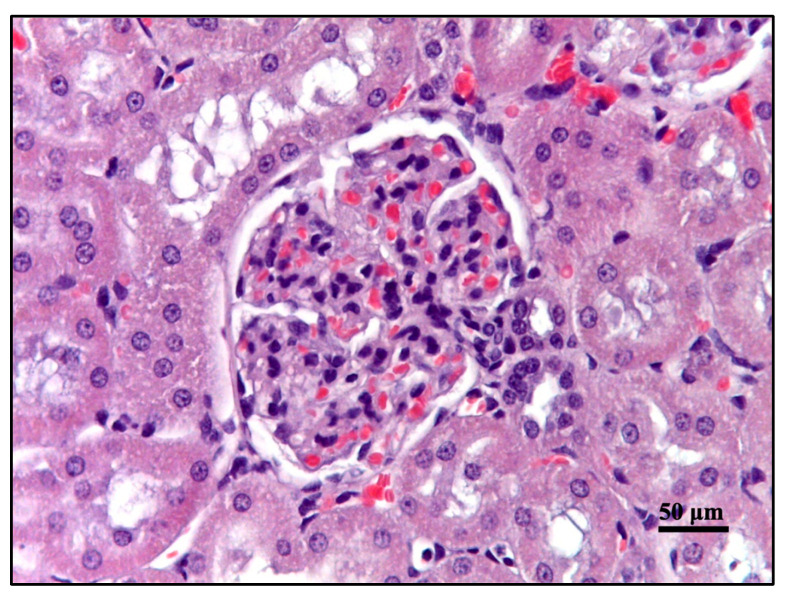
A glomerulus of a rat from the control group; stain—hematoxylin and eosin, magnified ×400.

**Figure 7:**
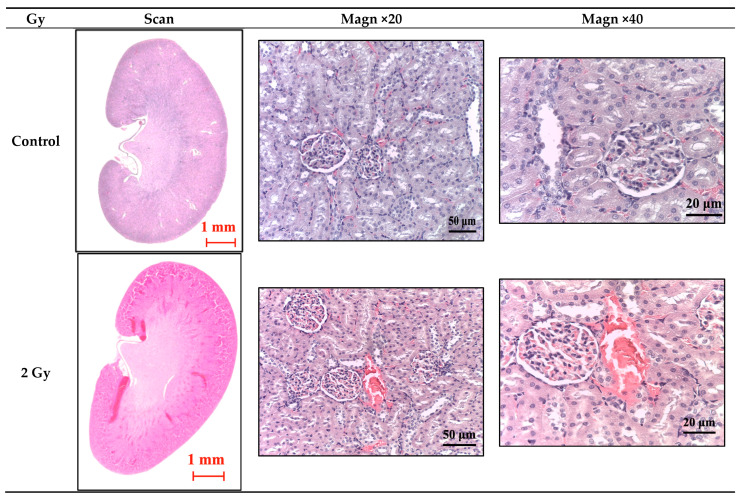

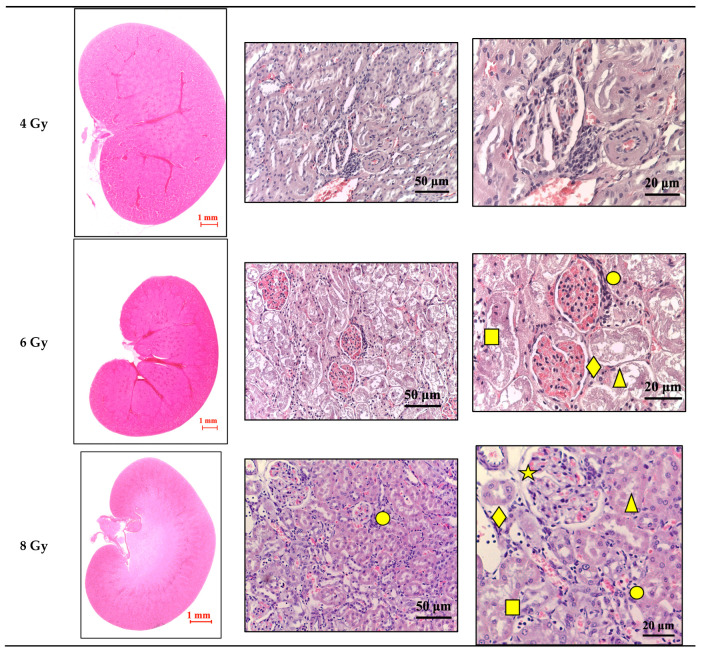
Kidneys of rats from experimental groups at different radiation doses, evaluated 7 days post-irradiation; stain—hematoxylin and eosin; different magnification. On the slides: dilation of Bowman’s capsule (*), vacuolization (∆), dystrophic changes in nephron tubules (**□**), perivascular and periglomerular edema (◊), mild inflammatory (●).

**Figure 8:**
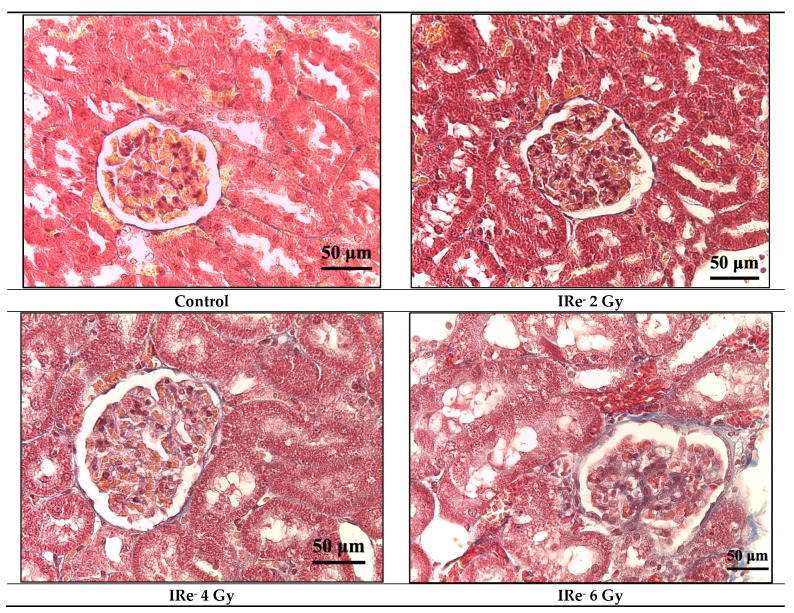
Kidneys of rats from experimental groups at different radiation doses, evaluated 7 days post-irradiation; stain—Masson’s trichrome; magn. ×40.

Light microscopy of kidney sections from the control group (intact animals) revealed normal histoarchitecture: the renal tubules were appropriately arranged within the cortex, including both proximal and distal nephron tubules, while in the medulla, the remaining segments of the nephron were observed (Figure 6 and Figure 7). In animals subjected to a single dose of irradiation at 2 Gy, 4 Gy, 6 Gy, or 8 Gy, significant morphological alterations were detected in the renal structures, particularly affecting the glomerular endothelium and the epithelial cells of both proximal and distal nephron tubules. The severity of these changes exhibited a clear dose-dependent pattern (Figure 8). Additionally, a summary of these morphological changes is presented in Table 2.

In the groups exposed to 2 Gy, 4 Gy, and 6 Gy of irradiation, there were significant dose-dependent pathomorphological changes, primarily characterized by an increase in the number of congested blood vessels and a corresponding rise in the vascularization index (Figure 6 and Figure 7). Specifically, kidney tissues exposed to a 2 Gy dose exhibited a marked vascular reaction, with a 14.2% increase in the number of small, congested vessels. In the 4 Gy irradiation group, these pathological changes persisted, with a slight further increase to 22.2%. The most pronounced vascular congestion, indicated by a 62.2% increase in the number of both small and large blood vessels, was observed in the group exposed to a 6 Gy dose. The degree of severity of fibrotic changes in the kidney depended on the dose of exposure to electrons in trichrome reactions according to Masson (Figure 8).

**Figure 9:**
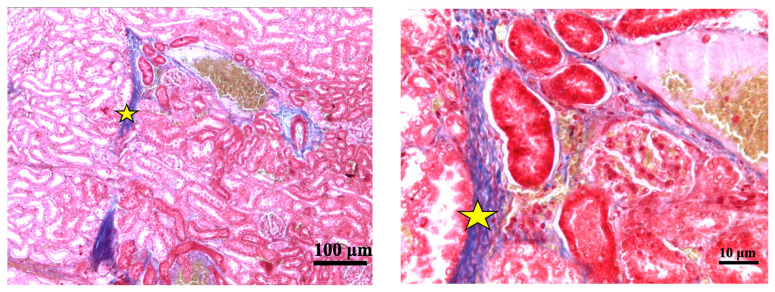
The kidney of a rat from 8 Gy group (7 days post-irradiation); stain—Masson’s trichrome; magn.: left ×100, right ×200. On the slides: mild fibrosis (*).

In the 8 Gy irradiation group, histological examination revealed several significant alterations, including dilation of Bowman’s capsule, vacuolization, and atrophy of certain nephron tubules (Figure 7). Additionally, perivascular and periglomerular edema, dystrophic changes, and mild inflammatory signs were noted. These alterations were markedly more pronounced in the 8 Gy group, with focal fibrosis and more severe tissue damage being particularly evident. The share of damaged glomeruli in the 8 Gy group was substantial, with the area of the glomerulus with Bowman’s capsule measuring 5392.7 ± 838.2 μm^2^ (*p* < 0.05), accounting for up to one-sixth of the kidney tissue. Additional findings included pycnotic changes in the nuclei of proximal tubule cells, dissociation of macula dense cells, and pronounced perivascular and periglomerular edema. The most pronounced fibrotic changes in the kidney were found after exposure to electrons at a dose of 8 Gy in Masson’s trichrome reactions (Figure 8 and Figure 9).

### 4.4. TUNEL Assay

**Figure 10:**
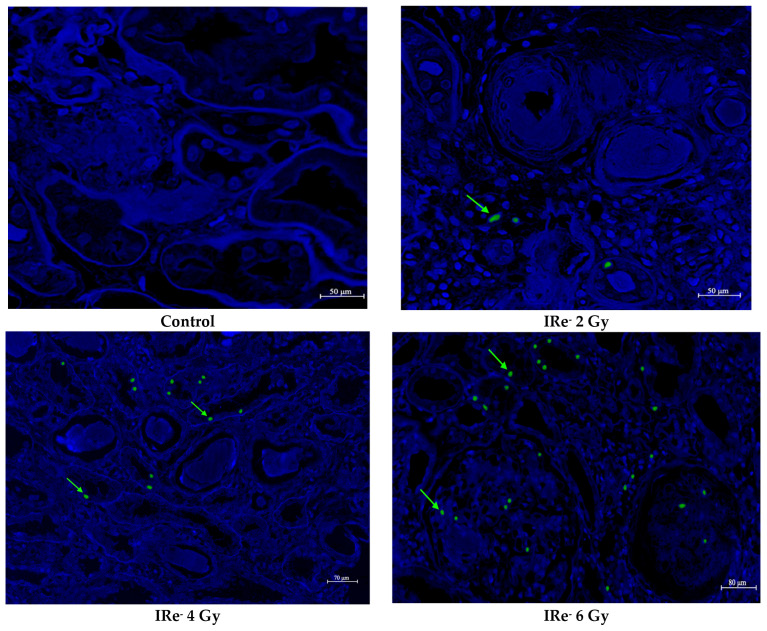

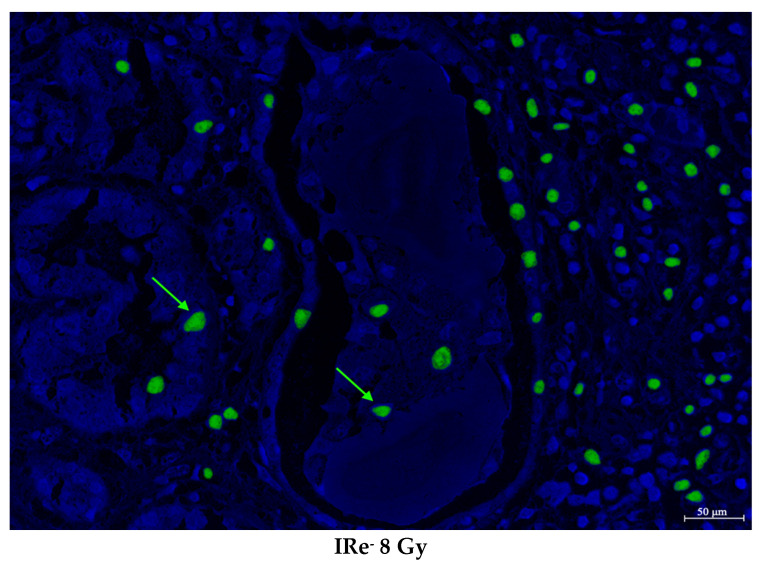
TUNEL staining of kidney tissue of all experiment groups (7 days post-irradiation): TUNEL-positive cells (green, pointers are green arrows); DAPI-positive cells (blue cells); scale bar = 50 μm, 70 μm and 80 μm.

**Figure 11:**
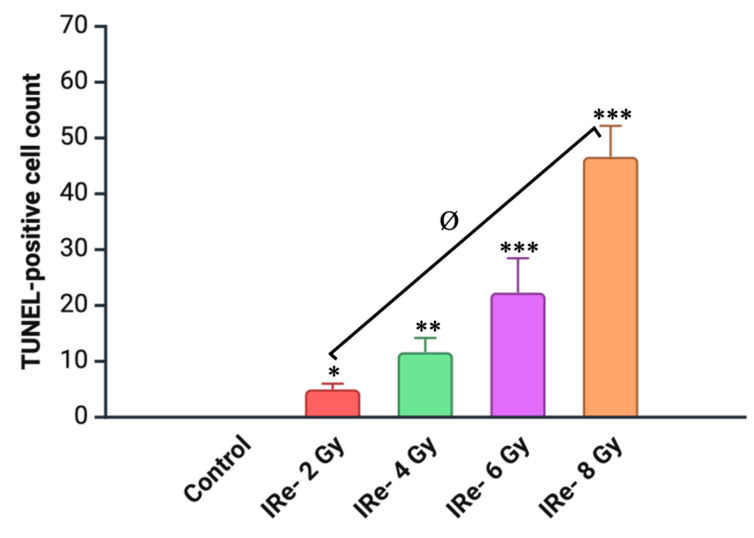
Quantitative distribution of TUNEL-positive cells in kidney tissue sections after electron irradiation (7 days post-irradiation). Data are presented as mean ± SD. Experimental groups are numbered according to the study design. Statistically significant differences are indicated by symbols: *—comparison with control group (*p* < 0.05); **—comparison with control group (*p* < 0.01); ***—comparison with control group (*p* < 0.001); ø—comparison between group II (2 Gy electron dose) and group IV (8 Gy electron dose) (*p* < 0.001).

To evaluate the impact of electron irradiation on apoptotic cell death in renal tissues, we conducted a TUNEL assay across all experimental groups (control, 2 Gy, 4 Gy, 6 Gy, and 8 Gy) (Figure 10 and Figure 11). The TUNEL assay revealed a significant increase in TUNEL-positive renal tubular epithelial cells in the irradiated groups compared to the control group. The degree of apoptosis was dose-dependent, with the 2 Gy and 4 Gy groups showing moderate increases in apoptotic cells, while the 6 Gy and 8 Gy groups exhibited a more pronounced elevation in TUNEL-positive cells, indicating a progressive increase in apoptosis with higher doses of electron irradiation.

### 4.5. ICH Analysis

**Figure 12:**
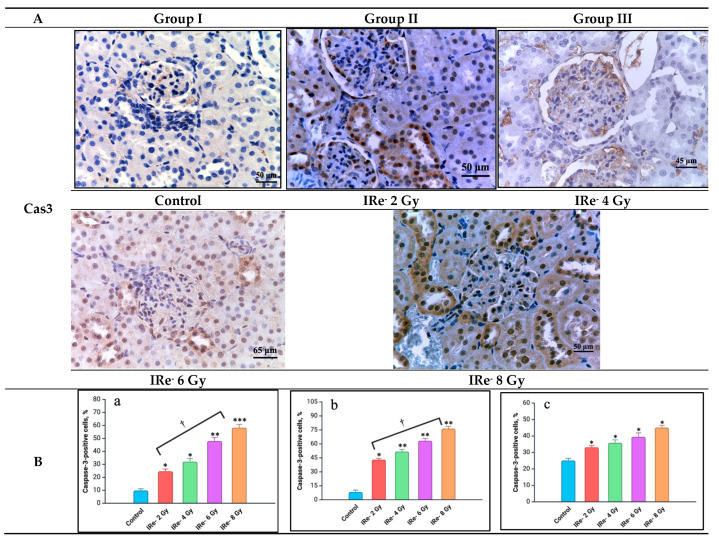
Kidneys from the control and experimental groups: (**A**)—immunohistochemical reactions with antibodies to caspase-3, magnification ×40; scale bar—45 μm, 50 μm, 65 μm; (**B**)—quantification of caspase-3-positive cells in renal tissue according to the immunohistochemical analysis, graph: (**a**)—caspase-3-positive cells in the renal medulla; (**b**)—caspase-3-positive cells in the proximal and distal tubules of nephrons; (**c**)—in the tubules of the loop of Henle and the collecting ducts. Experimental groups are numbered according to the study design. All data are presented as mean ± SD. Statistically significant differences are indicated by symbols: *—comparison with control group (*p* < 0.05); **—comparison with control group (*p* < 0.01); ***—comparison with control group (*p* < 0.001); †—comparison between group II (2 Gy) and group IV (8 Gy) (*p* < 0.01).

The immunohistochemical evaluation of apoptosis, specifically through the expression of caspase-3, revealed significant dose-dependent variations in the extent of cellular damage across different renal structures. Caspase-3-positive cells were identified in glomeruli (including endothelial cells, mesangial cells, and podocytes), renal corpuscles, epithelial cells of the proximal and distal tubules of nephrons, and the collecting ducts (Figure 12). The distribution and density of caspase-3-positive cells varied depending on the dose and specific location.

In the 2 Gy group, a moderate increase in caspase-3-positive epithelial cells in the proximal and distal nephron tubules was observed compared to the control group (*p* < 0.05). The glomerular endothelial cells also showed a slight, yet noticeable, increase in caspase-3 expression, though podocytes in Bowman’s capsule remained largely negative for caspase-3. In contrast, higher doses of irradiation, particularly in the 6 Gy and 8 Gy groups, led to a marked elevation in caspase-3-positive cells in all evaluated structures (*p* < 0.05), with the highest apoptotic activity detected in the 8 Gy group. This heightened expression likely reflects the cumulative effect of DNA damage, further promoting apoptotic cell death through the intrinsic mitochondrial pathway, as evidenced by the imbalance between pro-apoptotic and anti-apoptotic proteins.

Thus, these findings underscore the dose-dependent nature of radiation-induced apoptosis in kidney tissues, with caspase-3 serving as a reliable marker for evaluating the extent of apoptotic damage.

## 5. Discussion

In this experimental study, we investigated the effect of directed corpuscular beta irradiation (electrons) on the pelvic segment of rats, in particular on the kidney region. Electron energy of 10 MeV was administered at doses of 2 Gy, 4 Gy, 6 Gy, and 8 Gy. Given the paucity of literature on this topic, the aim of our study was to determine target doses that would allow a better understanding of the basic mechanisms of radiation-induced kidney damage and to create an experimental model for the possibility of further research in this area. In addition, this study was intended to lay the foundation for further in-depth studies in this area.

The high radiosensitivity of the kidneys makes them particularly susceptible to radiation-induced damage, emphasizing the importance of the present study. Our findings contribute significantly to the existing body of knowledge, enhancing our understanding of the pathophysiological changes induced by electron irradiation. Moreover, these results are crucial for developing effective strategies to mitigate the adverse effects of radiation therapy on the kidneys.

**Figure 13:**
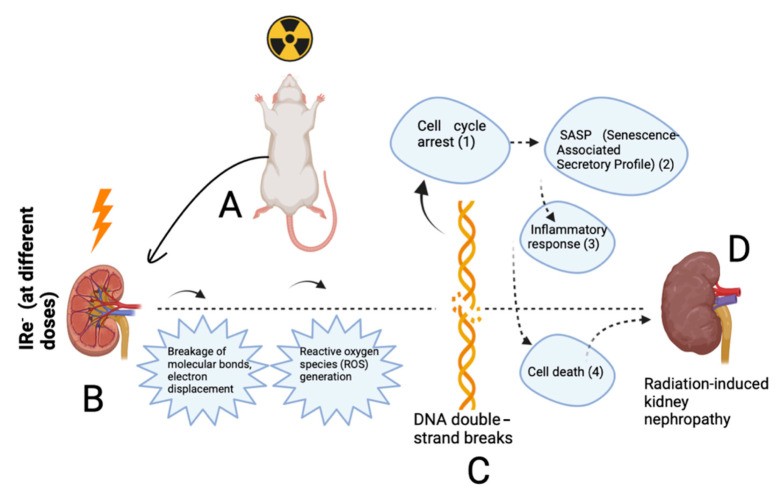
Illustration of the mechanism of radiation-induced nephropathy after electron irradiation (based on the specific literature): (**A**)—selection of animals for the study; (**B**)—irradiation of experimental animals using specialized facilities (different irradiation modes are possible); (**C**)—initiation of DNA double-strand breakdown; (**D**)—cascade of molecular and cellular reactions leading to direct disease formation (of varying severity).

Radiation therapy, particularly when using electron beams, presents a unique set of challenges and opportunities in clinical oncology. While electrons offer more localized energy deposition compared to gamma rays or X-rays, minimizing the damage to surrounding healthy tissues [25,26,27], the kidney’s high sensitivity to radiation demands careful consideration of dosing regimens. During the study of the specialized literature, we have compiled graphic material to facilitate the audience’s understanding of the main mechanisms of formation of radiation-induced nephropathy (Figure 13). It should be noted that while Figure 12 illustrates DNA double-strand breaks, this specific mechanism was not directly studied in our experiment; rather, our understanding of DNA double-strand breaks and their role in radiation-induced damage is based on a review of the relevant scientific literature [22,25,26,27,28]. This allows us to provide a conceptual framework, but further direct studies will be necessary to confirm the exact dynamics of DNA damage in this specific experimental setting.

The present results reveal that the kidneys exhibit dose-dependent progressive pathomorphological changes in histoarchitectonics following exposure to varying doses of electron irradiation. These findings align with previous studies, which have demonstrated the kidneys’ high vulnerability to radiation-induced injury, characterized by oxidative stress, inflammation, and apoptosis [10,28]. These changes are critical in the development of chronic radiation nephropathy, a condition that significantly impacts patient outcomes in cancer therapy. However, electron irradiation in our study resulted in specific morphological changes in the endothelium of vascular tubules and the epithelium of proximal and distal tubules of the nephron. Although our study did not directly compare the effects of different types of radiation, existing studies suggest theoretically that electron irradiation may result in less severe morphological damage compared to gamma or X-ray irradiation used in the same conditions [4,5,6]. Theoretically, this distinction is particularly important for clinical applications, where electron therapy may offer advantages in the treatment of retroperitoneal malignancies, potentially reducing the risk of radiation nephropathy and improving patient outcomes. Further studies are needed to quantify these morphological differences with different types of irradiations. And the present lannes should be interpreted with extreme caution.

An important consideration when using electron irradiation is the generation of secondary radiation, specifically Bremsstrahlung, which occurs as electrons decelerate upon interacting with tissue. Bremsstrahlung contributes to the radiation dose outside the primary treatment area, potentially increasing the risk of collateral damage to surrounding healthy tissues. However, due to the limited penetration depth of electrons compared to X-rays, the dose contribution from Bremsstrahlung in electron irradiation is generally lower. In this study, while the primary effects on renal tissue are attributed to direct electron interaction, some degree of tissue damage may result from Bremsstrahlung, particularly at higher doses of irradiation. Quantifying the relative contributions of electrons and Bremsstrahlung in renal damage requires further investigation. Nevertheless, current data suggest that electron-based therapies still offer a more localized and controlled radiation profile compared to conventional X-ray therapies.

The dose-dependent increase in pro-inflammatory cytokines (IL-1β, IL-6, and TNF-α) observed across all irradiated groups suggests an inflammatory response triggered by electron irradiation. This response is indicative of radiation-induced tissue damage, where reactive oxygen species (ROS) play a central role in initiating and propagating inflammation. The observed increase in cytokine levels corresponds with findings in studies of other radiation-induced organs, for example, liver disease [29], suggesting that similar molecular pathways may be involved in the pathogenesis of radiation nephropathy [30,31,32].

Interestingly, the anti-inflammatory cytokine IL-10 also showed a dose-dependent increase, which may reflect a compensatory mechanism aimed at mitigating the pro-inflammatory effects. However, the extent to which IL-10 fails to effectively counteract these pro-inflammatory responses, particularly at high doses, remains an area requiring further investigation [33,34]. Not excluded, the increase in IL-10 levels may act as a compensatory mechanism aimed at balancing this inflammatory response to prevent excessive damage. Such dynamics reflect the organism’s attempt to maintain homeostasis by controlling inflammation and responding to damage. At the same time, we do not exclude such a response due to the peculiarities of electron exposure.

Our study also demonstrated a significant increase in malondialdehyde (MDA) levels in all irradiated groups, indicating increased lipid peroxidation and oxidative stress. The increase in MDA levels correlated with the irradiation dose, suggesting that higher doses of electron irradiation lead to greater oxidative damage. Simultaneously, the observed decrease in the activity of key antioxidant enzymes such as superoxide dismutase (SOD) and glutathione (GSH) further confirms the presence of oxidative stress. This imbalance between ROS production and antioxidant capacity likely exacerbates tissue damage and aggravates and enhances the mechanisms of pathogenesis of radiation-induced kidney injury [35].

The TUNEL assay has proven to be a reliable method for the detection of DNA fragmentation, providing greater specificity in the identification of apoptotic cells. The results of the TUNEL assay further confirm the extent of kidney damage induced by electron irradiation, as evidenced by a significant increase in apoptotic cell death across all irradiated groups. The highest level of apoptosis was observed in the 8 Gy group, indicating that higher doses of electron irradiation result in more extensive cell death, likely due to the accumulation of irreversible DNA damage. This apoptotic response is closely associated with the activation of the caspase family of proteases, particularly caspase-3, which plays a pivotal role in executing apoptosis by degrading essential intracellular proteins [36,37]. Electron irradiation appears to trigger the intrinsic pathway of apoptosis, a process that is likely exacerbated by an imbalance between pro-apoptotic proteins such as Bax and anti-apoptotic proteins such as Bcl-2. These findings are consistent with previous studies demonstrating similar dose-dependent increases in apoptosis in response to other forms of radiation [38,39].

Our immunohistochemical analysis further supports these findings, revealing a marked increase in caspase-3-positive cells within the renal structures following electron irradiation. Caspase-3, a key marker of the terminal stage of apoptosis [36], was predominantly expressed in glomerular endothelial cells, podocytes, and tubular epithelial cells, reflecting the significant cytotoxic stress induced by irradiation. This increase in caspase-3 expression suggests a shift in the balance between cell proliferation and apoptosis, with a clear predominance of apoptotic processes. This shift towards apoptosis is particularly notable in higher-dose groups, aligning with our earlier TUNEL assay results and reinforcing the dose-dependent nature of electron-induced renal injury.

The present study identified distinct histopathological changes in kidney tissues following electron irradiation, notably an acute vascular reaction at lower doses (2 Gy, 4 Gy, 6 Gy) characterized by an increase in plethoric blood vessels without signs of fibrosis. Although the use of electron irradiation in treating kidney malignancies is not well documented, our findings align with similar studies on other radiation types. For example, exposure to 6 Gy of X-ray radiation has been shown to cause degenerative changes in the nephron epithelium, atypical glomeruli, and numerous blood vessels in the interstitial tissue [40]. Similarly, gamma rays at the same dose resulted in degeneration of the epithelium lining and numerous blood vessels [41]. Higher doses (8 Gy) of gamma rays have been associated with glomerulosclerosis and interstitial nephritis [42], and some studies have reported massive areas of necrosis and cystic transformation of nephron tubules [43,44].

In contrast, our study using a pulsed electron accelerator showed a dose-dependent increase in vascular changes without fibrosis at lower doses. However, at 8 Gy, fibrosis appeared, likely due to collagen synthesis by myofibroblasts in response to hypoxia from vascular congestion [45]. Interestingly, no necrotic changes were observed in the nephron tubules or glomeruli, which may be attributed to the softer, more localized effect of electron irradiation compared to gamma rays.

The absence of severe destructive changes might also be due to the short-term nature of our study. Previous research has shown that significant glomerular and tubular fibrotic changes can take months to develop after exposure to doses of 10 Gy [46] or higher [17,47]. Our assessment, conducted just one week after irradiation, may represent the early stages of damage, with more severe fibrotic changes possibly emerging over time.

The results of this study clearly demonstrate a dose-dependent response to electron irradiation in renal tissues, where increasing doses lead to progressively more severe molecular and histopathological changes. At lower doses (2 Gy and 4 Gy), we observed a moderate increase in oxidative stress markers such as MDA, accompanied by a slight decrease in antioxidant defenses, including SOD and GSH levels. These molecular alterations indicate an early onset of oxidative stress, which likely triggers the initial phase of cellular damage without causing immediate, extensive structural damage as observed histologically. This aligns with a mild inflammatory response, as evidenced by the modest elevation of pro-inflammatory cytokines IL-1β, IL-6, and TNF-α, and an increase in the anti-inflammatory cytokine IL-10, potentially reflecting an attempt to counterbalance the pro-inflammatory environment.

As the irradiation dose increased to 6 Gy, there was a marked escalation in oxidative stress, as indicated by further increases in MDA and a more significant reduction in antioxidant activity. The intensified oxidative stress likely exacerbates the inflammatory response, as demonstrated by the pronounced rise in pro-inflammatory cytokines, which, in turn, amplifies tissue damage. The observed increase in apoptotic cell death, particularly through the activation of the intrinsic pathway involving different groups of caspases (i.e., cascase-3), suggests that the accumulation of DNA damage is reaching a threshold where cellular repair mechanisms are overwhelmed, leading to apoptosis. This is, probably, supported by the imbalance between pro-apoptotic proteins like Bax and anti-apoptotic proteins such as Bcl-2, which facilitates the progression of apoptosis. These assumptions are based on the literature data on other types of irradiations [48,49,50,51], and further in-depth studies on the topic are needed to better understand these mechanisms for electron irradiation. Histologically, this corresponds to the observed acute vascular reactions and early signs of structural damage in renal tissues.

At the highest dose of 8 Gy, the molecular and cellular responses became significantly more pronounced, indicating a severe level of stress and damage. The near collapse of antioxidant defenses, coupled with the substantial increase in pro-inflammatory cytokines (especially, TNF-α), points to a state of oxidative and inflammatory overload. This environment is highly conducive to apoptosis, with the caspase’s cascade, particularly caspase-3, being fully activated, leading to extensive cell death. The corresponding histological findings, including significant vascular congestion, the onset of fibrosis, and widespread apoptotic cell death, reflect the culmination of these molecular processes. The lack of necrotic changes, despite the severity of the other damages, may be attributed to the more localized and potentially less penetrative nature of electron beams.

Overall, the correlation between the molecular and histopathological findings in this study highlights the complex interplay of oxidative stress, inflammation, and apoptosis in response to electron irradiation. The dose-dependent nature of these responses underscores the importance of carefully considering irradiation doses in clinical settings. Although electron beams appear to offer a more localized radiation therapy option with potentially reduced long-term damage compared to other types of radiation, the risk of significant renal injury at higher doses remains a critical consideration. Further research is warranted to explore protective strategies that could mitigate these effects, particularly focusing on enhancing antioxidant defenses and modulating inflammatory pathways to preserve renal function during radiation therapy.

## 6. Conclusions

This study investigated the effects of targeted corpuscular beta irradiation (electrons) on rat kidneys, revealing significant dose-dependent pathomorphological changes. These changes included increased oxidative stress, elevated levels of pro-inflammatory cytokines, and a marked increase in apoptotic cell death as revealed by TUNEL analysis. Notably, higher doses of electron irradiation were associated with the early stages of fibrosis formation and acute vascular reaction, suggesting the possibility of chronic radiation nephropathy.

Although electron beams have advantages in terms of energy localization, minimizing damage to surrounding healthy tissue, our results highlight the need for careful consideration of dosing regimens to protect renal function. The findings emphasize the importance of ongoing research into the long-term effects of electron irradiation, especially to better understand the progression of fibrosis and other chronic diseases. This study contributes to the growing body of knowledge needed to develop safer and more effective radiotherapy protocols, which will ultimately improve patient outcomes in clinical oncology.

## Figures and Tables

**Table 1:** Histological scale for assessing kidney damage (the analysis was performed on the 7th day after irradiation).

Criterion	Number of Points
Glomerulosclerosis	
None	0
<25% of the affected area	1
25–50% of the affected area	2
50–75% of the affected area	3
>75% of the affected area	4
**Focal necrosis of the glomerulus**	
None	0
1/3 of the affected area	1
2/3 of the affected area	2
>2/3 of the affected area	3
**Glomerular hypertrophy**	
None	0
>1	1
**Tubulointerstitial fibrosis**	
None	0
<10% of the affected area	1
10–25% of the affected area	2
25–50% of the affected area	3
50–100% of the affected area	4
**Necrosis of the epithelium of the nephron tubules**	
None	0
1/3 of the affected area	1
2/3 of the affected area	2
>2/3 of the affected area	3
**Congestion of blood vessels**	
None	0
<25% of the affected area	1
25–50% of the affected area	2
50–75% of the affected area	3
>75% of the affected area	4

**Table 2:** The results of the assessment of pathomorphological changes in the experimental groups.

Parameter	Group	Control	2 Gy	4 Gy	6 Gy	8 Gy
	Score					
**Glomerulosclerosis**	0	5	5	5	4	4
	1	0	0	0	1	0
	2	0	0	0	0	1
	3	0	0	0	0	0
	4	0	0	0	0	0
	AS	0.0 ± 0.0	0.0 ± 0.0	0.0 ± 0.0	0.2 ± 0.2	0.4 ± 0.4
**Focal necrosis of the glomerulus**	0	5	5	5	5	4
	1	0	0	0	0	1
	2	0	0	0	0	0
	3	0	0	0	0	0
	AS	0.0 ± 0.0	0.0 ± 0.0	0.0 ± 0.0	0.0 ± 0.0	0.2 ± 0.2
**Glomerular hypertrophy**	0	5	5	5	4	3
	1	0	0	0	1	2
	AS	0.0 ± 0.0	0.0 ± 0.0	0.0 ± 0.0	0.2 ± 0.2	0.4 ± 0.24
**Tubulointerstitial fibrosis**	0	5	5	5	3	1
	1	0	0	0	2	0
	2	0	0	0	0	1
	3	0	0	0	0	3
	4	0	0	0	0	0
	AS	0.0 ± 0.0	0.0 ± 0.0	0.0 ± 0.0	0.4 ± 0.24	2.2 ± 0.58
**Necrosis of the epithelium of the nephron tubules**	0	5	5	5	4	4
	1	0	0	0	1	0
	2	0	0	0	0	1
	3	0	0	0	0	0
	AS	0.0 ± 0.0	0.0 ± 0.0	0.0 ± 0.0	0.2 ± 0.2	0.4 ± 0.4
**Congestion of blood vessels**	0	5	3	2	1	4
	1	0	2	1	0	1
	2	0	0	2	2	0
	3	0	0	0	2	0
	4	0	0	0	0	0
	AS	0.0 ± 0.0	0.4 ± 0.4	1 ± 0.44	2 ± 0.55	0.2 ± 0.2

AS—average score.

## Data Availability

The study did not generate publicly available archival data.
